# Cutaneous Metastasis vs. Isolated Skin Recurrence of Invasive Breast Carcinoma after Modified Radical Mastectomy

**DOI:** 10.1155/2021/6673289

**Published:** 2021-02-16

**Authors:** Reza Hosseinpour, Mohammad Javad Yavari Barhaghtalab

**Affiliations:** ^1^Cancer institute, Imam Khomeini Hospital, Tehran University of Medical Sciences, Tehran, Iran; ^2^Department of General Surgery, Shahid Beheshti Hospital, Yasuj University of Medical Sciences, Yasuj, Iran

## Abstract

**Background:**

Five to ten percent of the patients with operable breast cancer develop a chest wall recurrence within 10 years following the mastectomy. One of the most distressing presentations of locally recurrent breast cancer is the appearance of cutaneous metastases. To the best of authors' knowledge, there is no study distinguishing skin metastasis from local recurrence, so the main aim of this report was to elucidate if these two features are important in the prognosis and management of the disease. *Case Presentation*. A 51-year-old woman referred to the breast clinic due to a painful mass in the left breast. The patient underwent the modified radical mastectomy (MRM) and left axillary lymph node dissection followed by 30 sessions of radiotherapy and 8 sessions of chemotherapy (T3N1M0, ER−, and HER2+). About 15 months after the surgery, she presented with redness and eruptive lesions over the mastectomy scar that increased in size within a three-month follow-up.

**Conclusion:**

Mastectomy is not an absolute cure in the treatment of an invasive breast cancer because almost always, there is a recurrence risk and possibility of metastasis. It is vital to differentiate between local recurrence and skin metastasis because it would alter the overall treatment decision, prognosis, and patient outcomes.

## 1. Background

Five to ten percent of patients with operable breast cancer develop a chest wall recurrence within 10 years following the mastectomy [1]. One of the most distressing presentations of locally recurrent breast cancer is the appearance of cutaneous metastases [2]. It usually presents as a firm nodule at the site of the primary cancer that can be associated with ulceration, bleeding, and pain [3]. Cutaneous breast metastases most commonly present on the chest wall [4]. The abdomen, back, head and neck, scalp, and upper extremities are also common sites [5, 6]. Cutaneous metastases show that the underlying tumor has infiltrated into the skin, blood capillaries, and lymph vessels [1]. The presence of skin metastases signifies widespread systemic disease and a poor prognosis [6]. To the best of authors' knowledge, there is no study distinguishing skin metastasis from local recurrence, so in this study, our main concerns are if we could distinguish the skin lesions which are presented after the mastectomy as cutaneous metastasis or isolated skin recurrence, and if we could consider these lesions as metastasis or recurrence, it would change the prognosis or management of the disease.

## 2. Case Presentation

A 51-year-old woman referred to the breast clinic due to a painful bulging in the left breast. The patient had no past medical history and family history of any cancer within her family. On the initial presentation, she had two palpable, 6 × 4 cm and 4 × 2 cm, well-circumscribed hard masses at the upper outer quadrant of the left breast with no skin changes or attachment to the underlying muscle. The left axilla had several palpable lymph nodes with the maximum diameter of 2 cm. Core needle biopsy of the breast mass revealed an invasive ductal carcinoma, estrogen and progesterone receptors (ER/PR) negative, human epidermal growth factor receptor 2 (HER2) positive 3+, and Ki-67 25% nuclear labeling. The patient underwent the modified radical mastectomy (MRM) and left axillary lymph node dissection followed with 30 sessions of radiotherapy and 8 sessions of chemotherapy. Pathological examination of the mastectomy specimen revealed a 6 cm tumor nodule with the invasive ductal carcinoma, solid and comedo type, grade 2, ER/PR negative, and HER2 positive. All surgical margins were uninvolved by the tumor, and 16 out of 19 lymph nodes were involved by invasive carcinoma (T3N1M0, ER−, and HER2+).

**Figure 1:**
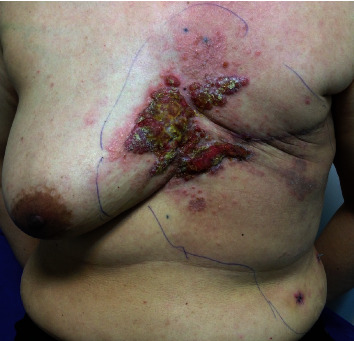
Indurated erythematous ulcerative papulonodular plaque appearance lesion on the previous MRM scar.

About 15 months after the surgery, she presented with redness and eruptive lesions over the mastectomy scar that increased in size within a three-month follow-up in the clinic. On the physical examination, the skin lesion was a localized, well-defined, 7 × 5 cm, indurated erythematous ulcerative papulonodular plaque on the left anterior chest and also abdominal wall over the previous MRM scar (Figure 1).

An incisional biopsy of the skin from the MRM site was taken and was sent for pathology evaluation. The patient was revealed to have an invasive ductal carcinoma with apocrine features with invasion to the papillary dermis (grade three or high grade), ER/PR negative, HER2 positive 3+, Ki-67 15%, and E-cadherin positive. The patient underwent whole-body bone scintigraphy or technetium 99m-methyl diphosphonate (Tc99m-MDP) and spiral chest, abdomen, and pelvic CT-scan with intravenous (IV) contrast, and both studies had normal findings. The patient received 24 sessions of chemotherapy afterward.

## 3. Discussion

Recovery or recurrence is the natural history of a patient with breast cancer who undergoes the mastectomy procedure. Recurrence occurs as local or local and distant metastases. Local isolated skin recurrence without concomitant metastatic disease after the mastectomy has been rarely reported [7]. More than 20% of all cutaneous metastases are arising from the breast cancer, and this is more than any other malignancies in women. If a patient has skin metastases, a systemic disease might be inevitable, and it shows a poor prognosis [6, 8]. The most common sites which could be involved are the chest wall, the abdomen, the back, the head and neck, and the upper extremities [5, 6, 8].

It has been shown that a small percentage of local recurrences after treatment for ductal carcinoma in situ (DCIS) has a component of invasive disease, which may increase the risk of distant metastases and have a reduced rate of survival [9]. Young patient age, incomplete breast tissue resection (residual breast tissue and inadequate margin resection during surgery), presence of occult invasive focus, size of the tumor, multiquadrant tumors, comedo type and high nuclear grade, and diffuse necrosis are reported as risk factors for local recurrence [10].

It is difficult to cure an advanced metastatic breast cancer as there may be an ultimate resistance to cytotoxic treatment. Progression of cutaneous metastases to a fungating ulcerative mass could destruct psychological well-being, grow social isolation, and reduce the quality of life [8]. In the advanced stages of the breast cancer with cutaneous metastasis, it is not possible to do the surgery; so, limited surgeries are done, and this is the reason for why the recognition of breast cancer at an early stage is very important for the therapeutic approach [8].

If locoregional recurrence occurs within the same quadrant as the initial tumor, it does not affect the outcome [11]. On the contrary, diffuse locoregional recurrence involving the breast skin or presenting as axillary recurrence has a poorer prognosis than isolated breast involvement [12]. Patients with locoregional recurrence involving the skin have a 50% rate of local failure compared with only a 14% failure rate in patients without skin involvement [13]. Skin involvement at locoregional recurrence increases the incidence of distant metastases from 14% to 44% [14]. It has also been shown that patients with locoregional recurrence involving the skin have a 44% to 83% risk for distant metastases simultaneously or within 2 months of locoregional recurrence, compared with 5% for those patients without skin involvement at recurrence [15, 16]. Locoregional recurrence involving the skin portends a poor overall survival of 13% to 18% between 5 and 10 years after recurrence [13, 17].

Mastectomy is not an absolute cure in the treatment of an invasive breast cancer because almost always, there is a recurrence risk and possibility of metastasis. As it is impossible to perform the surgery, if the breast cancer progresses to the advanced stages with cutaneous metastasis, the recognition and management of the disease at earlier stages are very important, so it is vital to differentiate between local recurrence and skin metastasis because it would alter the overall treatment decision, prognosis, and patient outcomes.

## Data Availability

The datasets used and/or analyzed during the current study are available from the corresponding author upon reasonable request.

## Abbreviations

MRM: Modified radical mastectomy
ER/PR: Estrogen and progesterone receptors
CT-scan: Computed tomography scan
IV: Intravenous contrast
DCIS: Ductal carcinoma in situ
HER2: Human epidermal growth factor receptor 2
Tc99m-MDP: Technetium 99m-methyl diphosphonate.
